# Bone Marrow-Derived Mesenchymal Stem Cells Exert Diverse Effects on Different Macrophage Subsets

**DOI:** 10.1155/2018/8348121

**Published:** 2018-07-24

**Authors:** Bin Chen, Yanhong Ni, Jiaying Liu, Yangheng Zhang, Fuhua Yan

**Affiliations:** ^1^Department of Periodontology, Nanjing Stomatological Hospital, Medical School of Nanjing University, Nanjing 210008, China; ^2^Center Laboratory, Nanjing Stomatological Hospital, Medical School of Nanjing University, Nanjing 210008, China

## Abstract

Mesenchymal stem cells (MSCs) and their secreted molecules have shown great potential for tissue regeneration and the treatment of inflammation and autoimmune diseases. However, they can also be associated with therapeutic failure or even side effects. Possible causes for this could include the state of the stem cells themselves and the influence of the local microenvironment, wherein macrophages play important roles. As such, we utilized conditioned medium from bone marrow-derived MSCs (MSC-CM) and studied its effect on different macrophage subsets. Effects on macrophage proliferation, apoptosis, polarization, and phagocytosis were determined, and it was discovered that MSC-CM had no significant effect on macrophage proliferation but inhibited M0 macrophage apoptosis and marginally induced M1 macrophage apoptosis. MSC-CM was shown to reduce CD80 expression on the surface of M1 macrophages. Moreover, it promoted and inhibited CD163 expression on the surface of M0 and M1 macrophages, respectively. However, MSC-CM tended to initially promote CD163 expression on M2 macrophages but inhibited expression of this marker after additional incubation time. Unlike MSCs, MSC-CM had no significant effect on the expression of TNF-*α* and IL-10 in macrophages. Thus, the effect of MSC-CM on different types of macrophages is different, and after stem cells are implanted, their effects on the local immune microenvironment are closely related to the original immune status of the implantation site. Therefore, we suggest that when utilizing stem cells for therapeutics, the immune status of the treatment site should be fully elucidated.

## 1. Introduction

In recent years, mesenchymal stem cells (MSCs) have received remarkable attention for tissue regeneration and the treatment of inflammation and autoimmune diseases; they have shown good potential for application, but failure and even side effects have been reported in some clinical trials [1–3]. Although hypotheses regarding differences in treatment outcomes do not rule out heterogeneity and the immunogenicity of MSCs, the characteristics of treated sites have also not been overlooked. The microenvironment of the treatment site itself, especially the immune microenvironment, might play an important role in treatment efficacy [4–7].

Given the complexity of the immune system and the important role of macrophages in inflammation and regeneration [6, 8–10], we selected macrophages, the participants and regulators of immune system, as the basis of our research. Because of their functional diversity, macrophages play critical roles in immune regulation, development, and tissue remodeling [11]. Simultaneously, macrophages are the most plastic cells and are usually divided into two phenotypes including classical activated M1 macrophages and alternatively activated M2 macrophages [12].

It is becoming clear that it is necessary to have a deeper understanding of the interaction between MSCs and the host immune system after MSC implantation. Indeed, many studies have focused on the effects of MSCs on macrophages [13–15]; however, it is worth noting that macrophages are highly plastic and exist as different subtypes [16]. Therefore, it is essential to better understand the effects of MSCs on different subtypes of macrophages to provide solid theory as the basis for clinical treatment.

Therefore, to preliminarily explore the causes of stem cell therapy failure that occurs in some cases, we utilized MSC conditioned media (MSC-CM) (to rule out the immunogenicity of stem cells themselves and avoid complex cell–cell cross-talk) and macrophages (participants in innate immunity, cells that link innate and specific immunity, and regulators of specific immunity) to investigate the effects of MSC-associated molecules on the biological behavior of macrophages and to explore changes in the local immune microenvironment that occur after stem cell therapy.

## 2. Materials and Methods

### 2.1. Animals

Sprague-Dawley rats (male, 5-week-old) were obtained from the Sino-British SIPPPR/BK Lab. Animal Ltd. All animal care and experiments were performed under the institutional protocol approved by the Medical School of Nanjing University.

### 2.2. Cytokines and Reagents

Recombinant rat macrophage-colony stimulating factor (M-CSF), interferon-*γ* (IFN-*γ*), and interleukin-4 (IL-4) were purchased from PeproTech (Rocky Hill, NJ, http://www.peprotech.com). LPS from *Escherichia coli* 055:B5 was obtained from Sigma-Aldrich (St. Louis, MO, http://www.sigmaaldrich.com). Antibodies included FITC anti-rat CD11b/c and PE anti-rat CD80 (BioLegend, San Diego, CA, http://www.biolegend.com), Alexa Fluor® 647 anti-rat CD163 (Bio-Rad, US, http://www.bio-rad.com/), Annexin V-Alexa Fluor647/PI Apoptosis Assay Kit (FMSAV647-100, FcMAS, Nanjing, China, http://www.fcmacs.com/), inducible nitric oxide synthase (iNOS) (R&D, MN, https://www.bio-techne.com/), and Arginase 1 (Arg-1) (CST, https://www.cellsignal.com/).

Cell culture-related reagents included fetal bovine serum (FBS) and DMEM low sugar medium (glucose content 1 g/ml; Gibco, US, http://www.thermofisher.com/cn), as well as penicillin/streptomycin and PBS (Hyclone, US, http://www.thermofisher.com/cn).

### 2.3. Culture and Identification of Bone Marrow-Derived Macrophages

Bone marrow-derived macrophages were obtained from 5-week-old male Sprague-Dawley rats. The tibiae and femurs were removed, and both ends were cut and washed with PBS to obtain bone marrow cells. Culture medium comprised DMEM containing 50 ng/ml of M-CSF, 10% FBS, 100 U/ml penicillin, and 100 *μ*g/ml streptomycin, which was used to induce bone marrow cell differentiation into macrophages and during the experiments (day 7–day 10); the concentration of M-CSF was reduced to 25 ng/ml, and there was no change in other components of the medium. Macrophage identification was performed using microscopic observation and flow cytometry to identify surface CD11b expression, as well as neutral red phagocytosis experiments.

### 2.4. Directional Induction of Macrophages

Macrophages were induced to transform into classically activated Ml macrophages using LPS + IFN-*γ*, with an LPS concentration of 100 ng/ml and an IFN-*γ* concentration of 30 ng/ml. IL-4 was used to induce the conversion of macrophages to surrogate activated M2 macrophages at a concentration of 10 ng/ml. Flow cytometry was used to detect the expression of CD80 and CD163 on the cell surface to initially verify directed activation. Western blot was used to detect the expression of iNOS and Arg-1 in the cells to further verify the M1 and M2 polarization.

### 2.5. Preparation of MSC-CM

BMMSC was purchased from AllCells, LLC (Emeryville, CA), and MSCs at passage 3-4 were used for this experiment. To prepare conditioned media, fresh medium was replaced when the cell density reached 40–50%; at the same time, control medium was prepared in the same way. Twenty-four hours later, the supernatant was collected, centrifuged at 3000 rpm/min for 10 min, and stored at −80°C. Both control medium and MSC-CM were used within 1 month of preparation. When used, media were thawed at 4°C.

### 2.6. Experiment Grouping

The six experimental groups were processed as indicated in Table 1.

### 2.7. Cell Viability Assay

According to the aforementioned grouping, macrophages were treated differently before performing this assay. After macrophages have been induced with LPS + IFN-*γ* and IL-4 for 24 hours, the corresponding culture medium was replaced according to these groups (Table 1), and after 24 h of cocultivation, cell viability was detected by the CCK-8 method (Dojindo, JP).

### 2.8. Analysis of Apoptosis

According to the previously mentioned experimental grouping, after 24 h of cocultivation, the digested cells were stained for annexin V and propidium iodide (PI) and detected by flow cytometry.

### 2.9. Cell Surface Staining and Flow Cytometric Analysis

According to the previously mentioned experimental grouping, the digested cells were stained for CD11b/CD80/CD163 after 24 h and 40 h of cocultivation, and the cell surface expression was detected by flow cytometry.

### 2.10. Phagocytosis Assays

According to the previously mentioned experimental grouping, after 24 h of cocultivation, the culture medium was replaced with PBS containing 0.05% neutral red (Sigma) for another 30–40 min. After the cells were sufficiently stained, they were washed. Neutral red staining was observed by light microscope. Cells were lysed with a 1 : 1 vol/vol solution of acetic acid/ethanol, and the absorbance was measured using a microplate reader (540 nm).

### 2.11. ELISA

For experiments studying the effect of bone marrow-derived MSC-CM on phagocytosis using different macrophage subtypes, supernatants preserved for the previously described experiments were used to detect the expression levels of TNF-*α* and IL-10 by ELISA (Neobioscience, China).

### 2.12. Statistical Analysis

Values are expressed as mean ± standard deviation (SD). One-way ANOVA with Tukey's post hoc analysis was applied to determine significance among more than two groups of parametric data. Analyses were performed using the GraphPad Prism software (version 6.01), and differences were considered significant at *p* < 0.05.

All experiments were repeated three times. The cells used for each of the three experiments were from different rats.

## 3. Results

### 3.1. Macrophage Induction and Identification

**Figure 1:**
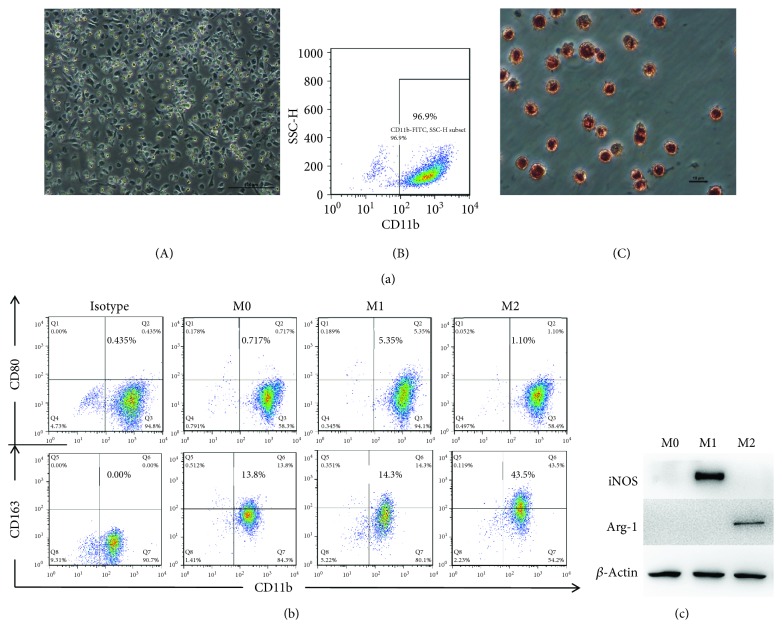
Identification of M1 and M2 macrophages. (a) (A) Morphology of macrophages as assessed by light microscopy on day 7 of cell culture (×100); (B) flow cytometry showing that the proportion of CD11b-positive cells was greater than 95% on day 7 of cell culture; (C) macrophages after phagocytic neutral red staining (×400). (b) Expression of CD80 and CD163 on macrophages after treatment with LPS + IFN-*γ* and IL-4 for 24 hours as assessed by flow cytometry. (c) Expression of iNOS and Arg-1 in macrophages after treatment with LPS + IFN-*γ* and IL-4 for 24 hours as assessed by Western blot.

During cell culture, microscopic observation revealed that bone marrow-derived cell populations began to gradually adhere on the second day; on the fourth day, cell morphology was relatively stable and was a characteristic of that of macrophages. On the seventh day, flow cytometry was performed. The expression of CD11b on the cell surface was examined, and results showed that greater than 95% of cells were positive; moreover, based on neutral red assays, the cells exhibited good phagocytic activity (Figure 1(a)).

Unstimulated macrophages showed no expression of surface CD80, whereas those treated with LPS and IFN-*γ* exhibited increased expression of CD80 (Figure 1(b)) and iNOS (Figure 1(c)), which was consistent with the characteristics of M1 macrophages. Meanwhile, both surface CD163 expression and Arg-1 expression were increased on IL-4-treated macrophages (Figures 1(b) and 1(c)), consistent with the characteristics of M2 macrophages.

### 3.2. Effect of MSC-CM on Viability and Apoptosis of Different Macrophages

#### 3.2.1. Effect of MSC-CM on Viability

**Figure 2:**
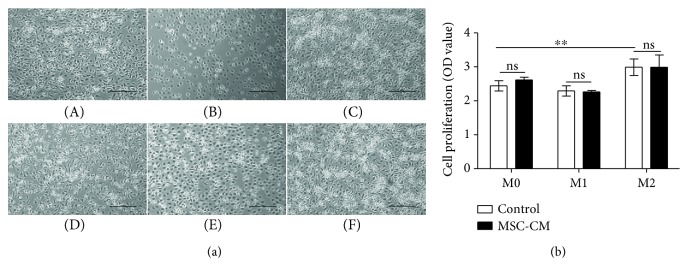
Effect of mesenchymal stem cell conditioned medium (MSC-CM) on the proliferation of different types of macrophages (results after 24 hours of coculture of different types of macrophages with MSC-CM or control medium). (a) Macrophages were observed by light microscopy (×100) after performing different treatments as follows: M0 (A); M1 (B); M2 (C); M0 + MSC-CM (D); M1 + MSC-CM (E); M2 + MSC-CM (F). (b) The effect of MSC-CM on the proliferation of different types of macrophages. ^∗∗^*p* < 0.01.

The proliferation of macrophages treated with LPS and IFN-*γ* decreased slightly, but there was no statistical difference. In contrast, the proliferation of macrophages treated with IL-4 significantly increased, and the difference was statistically significant (*p* < 0.01; Figure 2). Generally, MSC-CM had no significant effect on macrophage proliferation (*p* > 0.05; Figure 2).

#### 3.2.2. Effect of MSC-CM on Apoptosis

**Figure 3:**
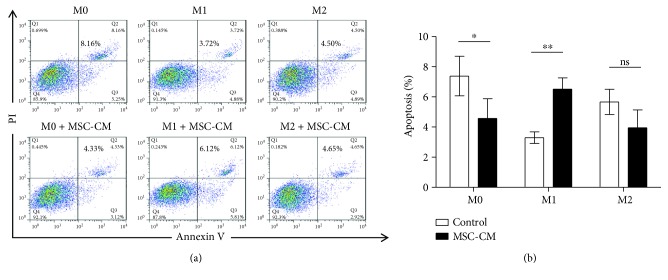
Effect of mesenchymal stem cell conditioned medium (MSC-CM) on apoptosis in different types of macrophages. (a) Different subsets of macrophages (M0, M1, and M2) were left untreated or treated with MSC-CM for 24 hours, stained with propidium iodide (PI) and annexin V, and subjected to flow cytometry. (b) Quantification of apoptosis and statistical analysis; ^∗^*p* < 0.05, ^∗∗^*p* < 0.01.

MSC-CM slightly inhibited the apoptosis of M0 macrophages (*p* < 0.05; Figure 3) and promoted M1 macrophage apoptosis (*p* < 0.01; Figure 3) without significantly affecting the apoptosis of M2 macrophages (*p* > 0.05; Figure 3).

### 3.3. Effect of MSC-CM on Macrophage Polarization

**Figure 4:**
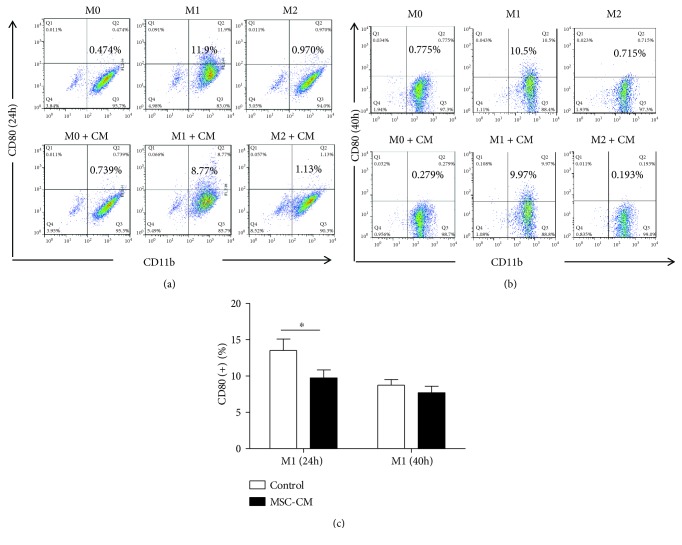
Effect of mesenchymal stem cell conditioned medium (MSC-CM) on the expression of CD80 on different macrophage subtypes. Expression of CD80 on the surface of macrophages after coincubation with MSC-CM for 24 h (a) and 40 h (b), as assessed by flow cytometry. (c) Quantification of CD80 expression and statistical analysis; ^∗^*p* < 0.05.

**Figure 5:**
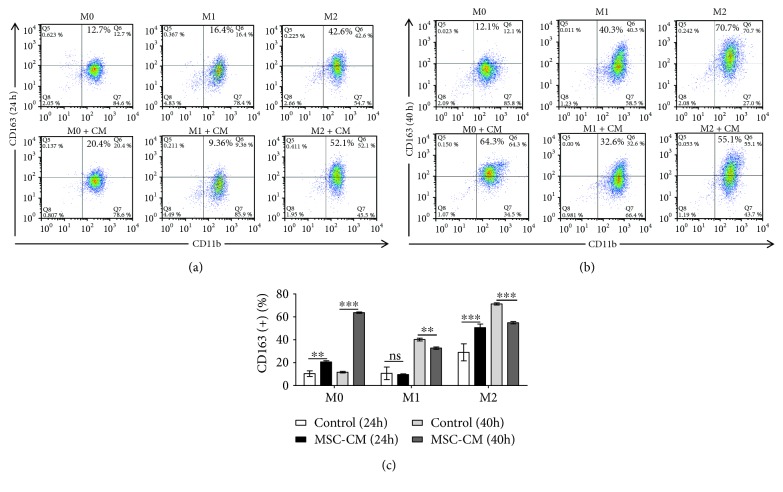
Effect of mesenchymal stem cell conditioned medium (MSC-CM) on the expression of CD163 on different macrophage subtypes. Expression of CD163 on the surface of macrophages after coincubation with MSC-CM for 24 h (a) and 40 h (b), as assessed by flow cytometry. (c) Quantification of CD163 expression and statistical analysis; ^∗∗^*p* < 0.01, ^∗∗∗^*p* < 0.001.

MSC-CM reduced the cell surface expression of CD80 on M1 macrophages after 24 h (*p* < 0.05; Figure 4) and promoted the expression of CD163 on the surface of M0 (*p* < 0.01; Figure 5), and this effect is more pronounced with time (*p* < 0.001; Figure 5). However, this treatment tended to increase the expression of CD163 on M2 macrophages after 24 h, after which, expression was inhibited (40 h; Figure 5).

### 3.4. Effect of MSC-CM on the Function of Different Macrophage Subtypes

#### 3.4.1. Effect of MSC-CM on Phagocytosis

**Figure 6:**
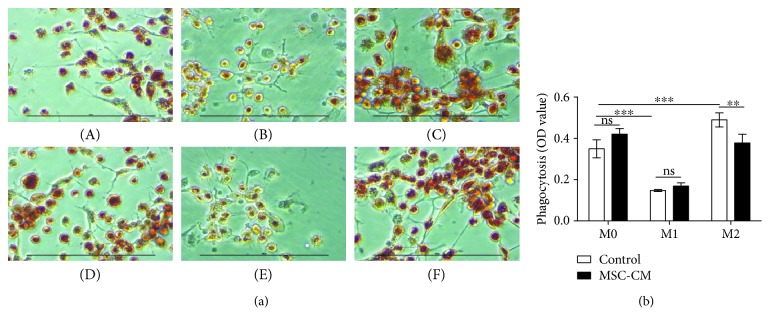
Comparison of phagocytic function of different macrophage subtypes after treatment with mesenchymal stem cell conditioned medium (MSC-CM) or not for 24 hours. (a) Phagocytic function was assessed by neutral red staining and light microscopy (×200, bar = 100 *μ*m) with the following groups: M0 (A); M1 (B); M2 (C); M0 + MSC-CM (D); M 1+ MSC-CM (E); M 2 + MSC-CM (F). (b) Quantification of effect of MSC-CM on phagocytic function of different types of macrophages; ^∗∗^*p* < 0.01, ^∗∗∗^*p* < 0.001.

Different macrophage subtypes have different phagocytic capabilities. Compared to that in unstimulated macrophages, the phagocytic ability of M1 macrophages was decreased, whereas that of M2 macrophages was increased (*p* < 0.001; Figure 6). MSC-CM inhibited the phagocytosis of M2 macrophages (*p* < 0.01; Figure 6) but had no significant effect on that of M1 macrophages (*p* > 0.05; Figure 6).

#### 3.4.2. Effect of MSC-CM on Secretory Function

**Figure 7:**
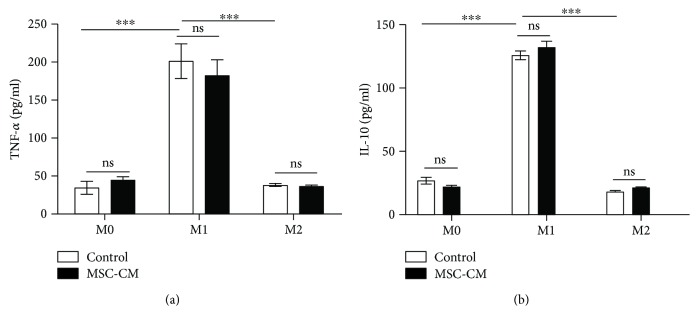
Effect of mesenchymal stem cell conditioned medium (MSC-CM) on the secretion of TNF-*α* (a) and IL-10 (b) by different subtypes of macrophages. Expression of these cytokines was assessed by ELISA after treatment of different macrophage subsets (M0, M1, and M2) with MSC-CM for 24 hours; ^∗∗∗^*p* < 0.001.

The ability of different macrophage subtypes to secrete TNF-*α* and IL-10 varies. TNF-*α* and IL-10 were significantly higher in M1 macrophages than in M0 and M2 macrophages (*p* < 0.001; Figure 7). However, MSC-CM had no significant effect on the ability of M1 and M2 subtypes to secrete TNF-*α* and IL-10 (Figure 7).

## 4. Discussion

Although the prospects for MSC therapy are promising, some in vivo studies have not achieved the desired results. For example, in a review by Marks et al., it was reported that even autologous stem cells, which are generally considered safe, still failed in the treatment of certain conditions including renal failure, systemic lupus erythematosus, and macular degeneration [2]. In postulating the reason for this, possible inherent flaws in the patient's stem cells were not ruled out; moreover, the complex role of the surrounding microenvironment was not excluded.

Numerous studies have confirmed the effectiveness of stem cell therapy for the treatment of periodontal defects in animals [17]. However, in a recent randomized controlled clinical study by Chen et al., stem cell therapy did not achieve better results [3]. The reasons for this might be as follows: (1) clinical periodontal defects are not often completely consistent, and the general condition of the patient, as well as the angle and depth of the defect, will affect the final results; in this case, a smaller sample size cannot detect statistically significant differences; (2) there are differences between humans and animals; and (3) the model used for most animal experiments is an immediate defect model, whereas in the study by Chen et al., the defects were caused by periodontitis—a chronic inflammatory disease; thus, the microenvironment surrounding stem cells could result in significant differences.

The results of this study show that MSC-CM has no significant effect on the proliferation of different macrophage subtypes; however, different effects on apoptosis were observed. Therefore, MSC-CM still influences the number of local macrophages. These cells are mainly derived from the proliferation of monocytes and native macrophages in tissues [18], whereas the latter is a marker of Th2 inflammation [19]. The steady state of local macrophages is very important for maintaining the local physiological state [20]. Our study shows that MSC-CM can reduce the proportion of M1 macrophages in local tissues by suppressing M0 apoptosis (*p* < 0.05; Figure 3) and promoting M1 apoptosis (*p* < 0.01; Figure 3), thereby inhibiting local inflammation. However, since this change was very small, its effect is thought to be limited.

Our results also showed that MSC-CM can slightly inhibit the phagocytic function of M2 macrophages, whereas the results of Jackson et al. showed that MSCs can promote macrophage phagocytosis [14]. This seems to be contradictory. However, careful analysis of the findings of Jackson et al. suggests that the associated mechanism is via the transmission of mitochondria; however, MSC-CM does not contain mitochondria, and the phagocytosis of M2 macrophages was stronger than that of M0 and M1 subsets, which require more energy, whereas the energy supply is lower in MSC-CM than control medium due to the consumption of MSC culture. Therefore, our results show that MSC-CM instead inhibits the phagocytic function of M2 macrophages.

In addition, our results also show that MSC-CM can slightly inhibit the expression of CD80 on the surface of M1 macrophages (*p* < 0.05; Figure 4), indicating that it has a specific role in inhibiting local inflammation. Interestingly, MSC-CM can significantly promote the expression of CD163 on the M0 macrophage surface, a trend that was consistent at 24 (*p* < 0.01; Figure 5) and 40 h of coculture (*p* < 0.001; Figure 5). Further, MSC-CM promoted the expression of CD163 on the surface of M2 macrophages at 24 h (*p* < 0.001; Figure 5); however, it inhibited this expression of CD163 at 40 h (*p* < 0.001; Figure 5). We know that CD163, also known as ED2 antigen, is a member of the scavenger receptor family in rats and might play a role in the activation of macrophages during hemolytic and/or inflammatory conditions [21]. CD163 was first identified as an endocytic receptor for hemoglobin-haptoglobin complexes. Later, it was identified as an erythroblast adhesion receptor, a receptor for tumor necrosis factor-like weak inducer of apoptosis; CD163 was also shown to bind both gram-positive and gram-negative bacteria and to act as an innate immune sensor and inducer of local inflammation [22]. Interestingly, CD163 also can trigger a signaling cascade leading to the secretion of signaling molecules, which implies that CD163 also acts as an immunomodulator [23]. Moreover, CD163+ macrophages promote angiogenesis and vascular permeability accompanied by inflammation during atherosclerosis [24]. In summary, the expression of macrophage CD163 can reflect its endocytosis, regulation of apoptosis, initiation and regulation of immunity, and its ability to promote tissue regeneration.

According to our results, MSC-CM not only fails to promote the expression of CD163 on the surface of M1 macrophages but also inhibits its expression (*p* < 0.01; Figure 5). In the case of periodontitis, which is associated with the dominant type of M1 macrophage [25], the application of MSCs reduces the expression of the original macrophage-associated CD163 in tissues, which is unfavorable for tissue repair. However, for inactivated M0 cells, MSC-CM can significantly increase the surface expression of CD163, and this effect increases over time. According to this result, MSC implantation indeed can promote tissue regeneration when there are many M0 macrophages in the microenvironment. More interestingly, MSC-CM promotes the expression of CD163 on the surface of M2 macrophages initially but later inhibits it, which indicates that when the proportion of tissue M2 macrophages is too high, MSCs will reduce the proportion of this subset. Through this mechanism, it is possible to prevent excessive tissue repair and scarring based on the fact that M2 macrophages are closely related to scar formation [26]. This might explain the findings of Otto et al.; specifically, upon injecting mesenchymal stem cells into nude mice with myocardial infarction, they found that stem cell therapy could reduce the infarct size, but no transdifferentiation to cardiomyocytes, endothelial cells, or smooth muscle cells was observed. This suggests that the therapeutic effects of MSCs are not directly related to differentiation into cells required for cardiac repair but rather occur through regulatory repair in this situation. It is worth noting that Otto et al. also showed that MSC-treated rats develop a much smaller scar area at 4 weeks than placebo-treated animals [27]. We know that scars indicate overrepair and that M2 macrophages are involved in this process; therefore, we could hypothesize that the reason as to why scars were smaller in the MSC therapy group might be that MSCs can prevent the M2 ratio from becoming too high in the tissue.

Finally, we also found that MSC-CM had no significant effect on the levels of TNF-*α* and IL-10 secreted by macrophages. This is inconsistent with the conclusion of Yin et al. that MSC can reduce the expression of TNF-*α* and promote IL-10 expression [28]. The reason may be that Yin et al. use MSC instead of MSC-CM. It is possible that MSCs have stronger immunomodulatory effects on macrophages than MSC-CMs, or there may have intercellular interactions between MSCs and macrophages. In addition, the macrophages used in the two studies are not the same, which may be the more important reason. Lacey et al. had compared M-CSF- and GM-CSF-induced macrophages from human and mouse and found that there are not only differences in the gene expression of macrophages in different species but also these genes respond differently to the inducing factors (M-CSF and GM-CSF) [29]. In addition, Raw264.7 is a macrophage cell line, and its acquisition does not require the induction of M-CSF or GM-CSF, given the fact that these very two factors will significantly affect the biological behavior of macrophages [29–31], which may also be the reason for the difference in the results of the two studies.

In summary, the effect of MSC-CM on different subtypes of macrophages was found to be distinct in vitro, and it is mainly reflected in its effect on nonactivated macrophages and weaker on macrophages that have been polarized. This result suggests that when performing stem cell therapy, the subtypes of macrophages in the treatment site should be identified. However, the in vivo environment is more complicated. Therefore, future research should confirm the impact of the immune microenvironment on stem cell therapy and its mechanisms on a more solid basis in vivo. What is more important is how to improve stem cell treatment effect by changing the local immune microenvironment.

## Figures and Tables

**Table 1:** Experimental groups used in the current study.

Group	LPS (100 ng/ml) + IFN-*γ* (30 ng/ml)	IL-4 (20 ng/ml)	MSC-CM volume/total culture medium volume^∗^100%
M0	−	−	0%
M1	+	−	0%
M2	−	+	0%
M0 + CM	−	−	50%
M1 + CM	+	−	50%
M2 + CM	−	+	50%

## Data Availability

The data that support the findings of this study are available from the corresponding author upon reasonable request.

## References

[1] Fisher S. A., Doree C., Mathur A., Taggart D. P., Martin-Rendon E. (2017). Cochrane corner: stem cell therapy for chronic ischaemic heart disease and congestive heart failure. *Heart*.

[2] Marks P. W., Witten C. M., Califf R. M. (2017). Clarifying stem-cell therapy’s benefits and risks. *New England Journal of Medicine*.

[3] Chen F. M., Gao L. N., Tian B. M. (2016). Treatment of periodontal intrabony defects using autologous periodontal ligament stem cells: a randomized clinical trial. *Stem Cell Research & Therapy*.

[4] Perry V. H., Brown M. C., Gordon S. (1987). The macrophage response to central and peripheral nerve injury. A possible role for macrophages in regeneration. *Journal of Experimental Medicine*.

[5] Li X. H., Yu X. Y., Lin Q. X. (2007). Bone marrow mesenchymal stem cells differentiate into functional cardiac phenotypes by cardiac microenvironment. *Journal of Molecular and Cellular Cardiology*.

[6] Wynn T. A., Vannella K. M. (2016). Macrophages in tissue repair, regeneration, and fibrosis. *Immunity*.

[7] Pajarinen J., Lin T., Gibon E. (2018). Mesenchymal stem cell-macrophage crosstalk and bone healing. *Biomaterials*.

[8] He L., Marneros A. G. (2013). Macrophages are essential for the early wound healing response and the formation of a fibrovascular scar. *American Journal of Pathology*.

[9] Bosurgi L., Cao Y. G., Cabeza-Cabrerizo M. (2017). Macrophage function in tissue repair and remodeling requires IL-4 or IL-13 with apoptotic cells. *Science*.

[10] Ben-Mordechai T., Holbova R., Landa-Rouben N. (2013). Macrophage subpopulations are essential for infarct repair with and without stem cell therapy. *Journal of the American College of Cardiology*.

[11] Guo J., Qiu X., Zhang L., Wei R. (2018). Smurf1 regulates macrophage proliferation, apoptosis and migration via JNK and p38 MAPK signaling pathways. *Molecular Immunology*.

[12] Das A., Sinha M., Datta S. (2015). Monocyte and macrophage plasticity in tissue repair and regeneration. *American Journal of Pathology*.

[13] François M., Romieu-Mourez R., Li M., Galipeau J. (2012). Human MSC suppression correlates with cytokine induction of indoleamine 2,3-dioxygenase and bystander M2 macrophage differentiation. *Molecular Therapy*.

[14] Jackson M. V., Morrison T. J., Doherty D. F. (2016). Mitochondrial transfer via tunneling nanotubes is an important mechanism by which mesenchymal stem cells enhance macrophage phagocytosis in the in vitro and in vivo models of ARDS. *Stem Cells*.

[15] Caires H. R., da Silva P. B., Barbosa M. A., Almeida C. R. (2018). A co-culture system with three different primary human cell populations reveals that biomaterials and MSC modulate macrophage-driven fibroblast recruitment. *Journal of Tissue Engineering and Regenerative Medicine*.

[16] Mosser D. M., Edwards J. P. (2008). Exploring the full spectrum of macrophage activation. *Nature Reviews Immunology*.

[17] Bartold P. M., Shi S., Gronthos S. (2006). Stem cells and periodontal regeneration. *Periodontology 2000*.

[18] Jenkins S. J., Ruckerl D., Thomas G. D. (2013). IL-4 directly signals tissue-resident macrophages to proliferate beyond homeostatic levels controlled by CSF-1. *Journal of Experimental Medicine*.

[19] Jenkins S. J., Ruckerl D., Cook P. C. (2011). Local macrophage proliferation, rather than recruitment from the blood, is a signature of TH2 inflammation. *Science*.

[20] Andrés V., Pello O. M., Silvestre-Roig C. (2012). Macrophage proliferation and apoptosis in atherosclerosis. *Current Opinion in Lipidology*.

[21] Polfliet M. M. J., Fabriek B. O., Daniëls W. P., Dijkstra C. D., van den Berg T. K. (2006). The rat macrophage scavenger receptor CD163: expression, regulation and role in inflammatory mediator production. *Immunobiology*.

[22] Fabriek B. O., van Bruggen R., Deng D. M. (2009). The macrophage scavenger receptor CD163 functions as an innate immune sensor for bacteria. *Blood*.

[23] Van Gorp H., Delputte P. L., Nauwynck H. J. (2010). Scavenger receptor CD163, a jack-of-all-trades and potential target for cell-directed therapy. *Molecular Immunology*.

[24] Guo L., Akahori H., Harari E. (2018). CD163+ macrophages promote angiogenesis and vascular permeability accompanied by inflammation in atherosclerosis. *Journal of Clinical Investigation*.

[25] Yu T., Zhao L., Huang X. (2016). Enhanced activity of the macrophage M1/M2 phenotypes and phenotypic switch to M1 in periodontal infection. *Journal of Periodontology*.

[26] Zhu Z. S., Ding J., Ma Z., Iwashina T., Tredget E. E. (2016). Systemic depletion of macrophages in the subacute phase of wound healing reduces hypertrophic scar formation. *Wound Repair and Regeneration*.

[27] Beitnes J. O., Øie E., Shahdadfar A. (2012). Intramyocardial injections of human mesenchymal stem cells following acute myocardial infarction modulate scar formation and improve left ventricular function. *Cell Transplantation*.

[28] Yin Y., Wu R. X., He X. T., Xu X. Y., Wang J., Chen F. M. (2017). Influences of age-related changes in mesenchymal stem cells on macrophages during in-vitro culture. *Stem Cell Research & Therapy*.

[29] Lacey D. C., Achuthan A., Fleetwood A. J. (2012). Defining GM-CSF– and macrophage-CSF–dependent macrophage responses by in vitro models. *Journal of Immunology*.

[30] Fleetwood A. J., Dinh H., Cook A. D., Hertzog P. J., Hamilton J. A. (2009). GM-CSF- and M-CSF-dependent macrophage phenotypes display differential dependence on type I interferon signaling. *Journal of Leukocyte Biology*.

[31] Fleetwood A. J., Lawrence T., Hamilton J. A., Cook A. D. (2007). Granulocyte-macrophage colony-stimulating factor (CSF) and macrophage CSF-dependent macrophage phenotypes display differences in cytokine profiles and transcription factor activities: implications for CSF blockade in inflammation. *Journal of Immunology*.

